# Electronic and Structural Disorder of the Epitaxial La_0.67_Sr_0.33_MnO_3_ Surface

**DOI:** 10.1021/acsami.3c17639

**Published:** 2024-04-15

**Authors:** Michael Verhage, Emma van der Minne, Ellen M. Kiens, Lucas Korol, Raymond J. Spiteri, Gertjan Koster, Robert J. Green, Christoph Baeumer, Cornelis F. J. Flipse

**Affiliations:** † Molecular Materials and Nanosystems (M2N)—Department of Applied Physics, Eindhoven University of Technology, Eindhoven 5612 AP, Netherlands; ‡ MESA+ Institute for Nanotechnology, Faculty of Science and Technology, University of Twente, Enschede 7522 NB, Netherlands; § Department of Physics & Engineering Physics, University of Saskatchewan, Saskatoon S7N 5A2, Canada; ∥ Department of Computer Science, University of Saskatchewan, Saskatoon S7N 5A2, Canada; ⊥ Stewart Blusson Quantum Matter Institute, University of British Columbia, Vancouver V6T 1Z4, Canada; # Peter Gruenberg Institute and JARA-FIT, Forschungszentrum Juelich GmbH, Juelich 52428, Germany

**Keywords:** LSMO, EPS, SPM, RXR, nonstoichiometry

## Abstract

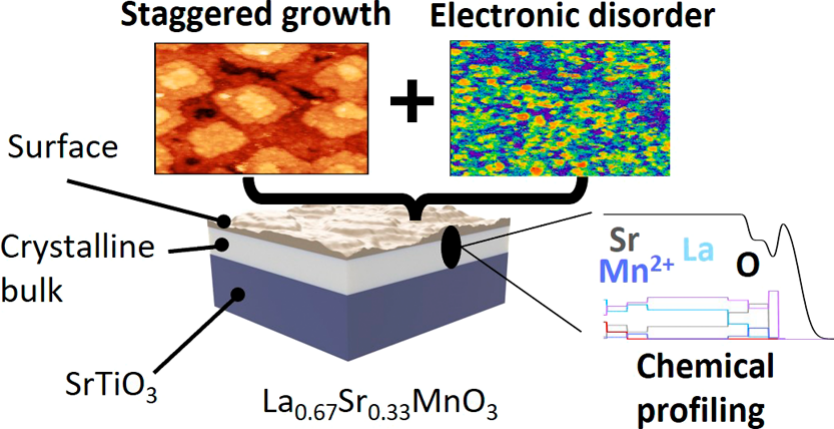

Understanding and tuning epitaxial complex oxide films are crucial in controlling the behavior of devices and catalytic processes. Substrate-induced strain, doping, and layer growth are known to influence the electronic and magnetic properties of the bulk of the film. In this study, we demonstrate a clear distinction between the bulk and surface of thin films of La_0.67_Sr_0.33_MnO_3_ in terms of chemical composition, electronic disorder, and surface morphology. We use a combined experimental approach of X-ray-based characterization methods and scanning probe microscopy. Using X-ray diffraction and resonant X-ray reflectivity, we uncover surface nonstoichiometry in the strontium and lanthanum alongside an accumulation of oxygen vacancies. With scanning tunneling microscopy, we observed an electronic phase separation (EPS) on the surface related to this nonstoichiometry. The EPS is likely driving the temperature-dependent resistivity transition and is a cause of proposed mixed-phase ferromagnetic and paramagnetic states near room temperature in these thin films.

## Introduction

Manganite complex oxides are considered to be attractive materials for spin-based devices^1−3^ due to a half-metallic character^4^ and high spin polarization.^5^ Doping the parent material LaMnO_3_ with Sr in La_0.67_Sr_0.33_MnO_3_ (LSMO) introduces insulator-to-metal and para-to-ferromagnetic transitions driven by a complex interplay of lattice structure, charge, and spin degrees of freedom.^6,7^ LSMO possesses an advantageous Curie temperature of up to 370 K^8^ that potentially enables spin control at room temperature. The control of these properties is of interest in fundamental electrochemical studies^9,10^ of manganites. LSMO has been used as a model catalyst to study the oxygen reduction and evolution reactions (ORR and OER, respectively) due to the tunability of the active manganese site and its promising bifunctional activity.^11−14^ A recent experiment has shown that active strain can be used to regulate the activity of LSMO thin films in the OER.^15^ This, combined with its high spin polarization, could be a useful tool for exploring the influence of electron spin on the OER.^16^

In addition to catalysis, and closely related to spin, LSMO has been extensively studied for phenomena such as colossal magnetoresistance^17,18^ and electroresistance.^19^ These phenomena are understood within the framework of electronic phase separation (EPS) and magnetic phase separation.^20^ Reports of chemical^20,21^ and pressure^22^ induced EPS of manganites are associated with bulk disorder and are essential to this family of metal oxides. A great deal of research has been done on LSMO thin films, exploring how changes in parameters such as substrate-induced strain (tensile or compressive), film thickness, substrate termination,^23^ and growth conditions affect structural, electronic, and magnetic behavior.^24−27^ However, there are still insights to be gained in the discrepancy between the surface structure and chemical composition and that of the bulk. To address this, we conducted both nanoscale imaging and spectroscopic analysis on an LSMO thin film to link nanoscale surface characteristics with those of the bulk to gain a comprehensive view of our LSMO films.

This work presents evidence that, for LSMO epitaxial thin films, both structural and chemical disorder persist at the surface, as revealed by high-resolution scanning probe microscopy (SPM) imaging. We observe nanometer-scale corrugation of the surface,^28^ consisting of the formation of a staggered growth of small islands,^29^ that deviates from an atomically smooth interpretation. We define an atomically smooth film as a surface roughness variation on the scale of atomic sizes with a correlation length on the order of the width of the vicinal steps. Pandya et al.^30^ have reported the emergence of such staggered growth or “wedding cake” structures, which are controlled by substrate-induced strain. Similarly, Kelley et al.^31^ have observed these islands in LSMO.

Furthermore, we used area-averaged chemical mapping by resonant X-ray reflectometry (RXR) to investigate a gradient in the chemical composition from a stoichiometric bulk to an excess of the Sr dopant near the surface. The chemical disorder is accompanied by a partial change in the valence state of Mn from Mn^3+^ to Mn^2+^ and an increase in oxygen vacancies.^32^ Sr segregation in the surface region was previously identified in ref (33) and linked to morphology restructuring.^28^

Using scanning tunneling spectroscopy (STS), we detected an EPS across the surface that is related to the nature of the staggered growth and chemical disorder. This relation between rough surface morphology and EPS has been reported for Ca-doped LaCaMnO_3_ films^34^ and thus appears to be a common feature of the manganite perovskite family.

The EPS and disorder are likely to be the cause of a magnetic phase separation in LSMO. Previous studies of Ca-doped manganites have reported the presence of ferromagnetic (FM) and paramagnetic (PM) areas that coexist and lead to percolation in charge transport.^22,35^ By studying the temperature-dependent resistance, a phenomenological model of phase separation was applied to differentiate between the FM and PM states,^35−37^ with a dominant FM phase at lower temperatures and a mixture of FM and PM at higher temperatures. A deeper understanding of the thin film LSMO surface enables insights into its applications in devices and catalysis.

## Results and Discussion

**Figure 1:**
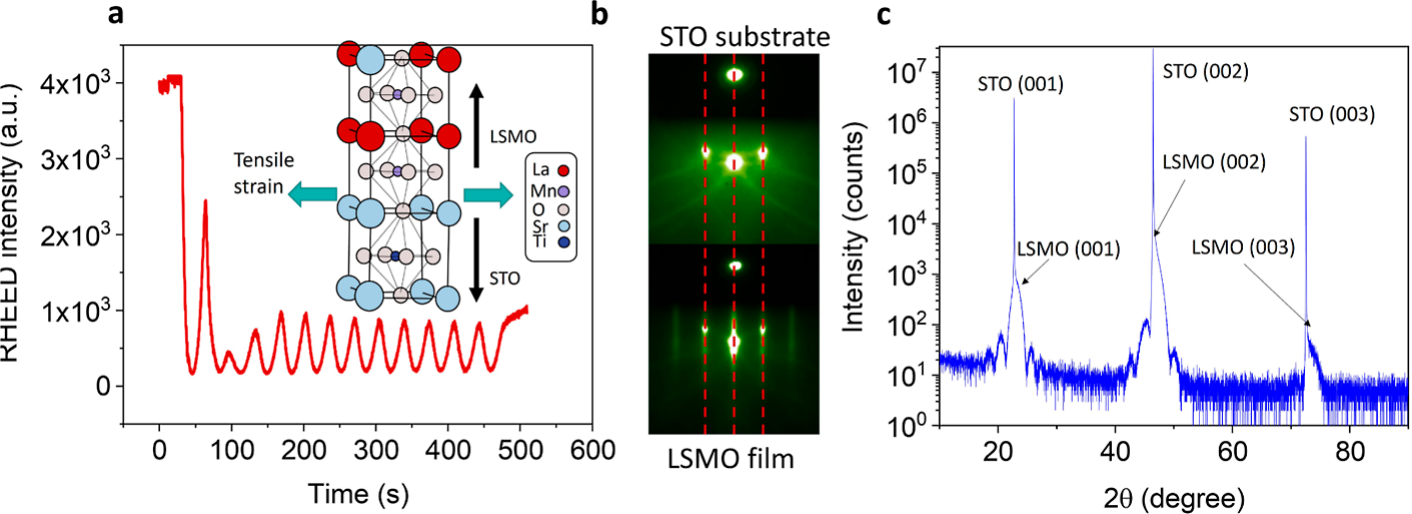
In situ growth characterization and structure of 13 u.c. LSMO films. (a) In situ RHEED intensity oscillations for a 13-unit cell (u.c.)-thick LSMO film grown on STO. Inset: schematic illustration of the atomic structure. (b) RHEED patterns of the substrate (top) and the film (bottom). The red dashed lines are a guide for the eye and indicate that the distance between the diffraction spots does not change. (c) Wide angle 2Θ–ω scan, indicating single (001)-oriented LSMO. From the position of the LSMO 002 peak a *c*-axis parameter of 0.385 nm is obtained.

LSMO films of 13 unit cell (u.c.) thickness were grown with pulsed laser deposition (PLD) on SrTiO_3_ (STO) 001 substrates; further experimental details are described in Methods. We used reflection high-energy electron diffraction (RHEED) to monitor growth in situ. The presence of clear RHEED intensity oscillations indicates layer-by-layer growth, as can be observed in Figure 1a. A schematic illustration of the crystal structure is shown in the inset of Figure 1a, and the growth on STO introduces tensile strain. A decrease in peak amplitude over time and faint streaks observed along with sharp diffraction spots in Figure 1b point toward the presence of disorder and roughness^38^ of the LSMO surface. The equal distance between the diffraction spots in the RHEED pattern, Figure 1b, indicates that the in-plane lattice parameters of the film match those of the substrate, implying the presence of tensile strain.^39^ The film quality was further verified by X-ray diffraction (XRD), indicating a pure (001) oriented LSMO phase, as seen in Figure 1c.

**Figure 2:**
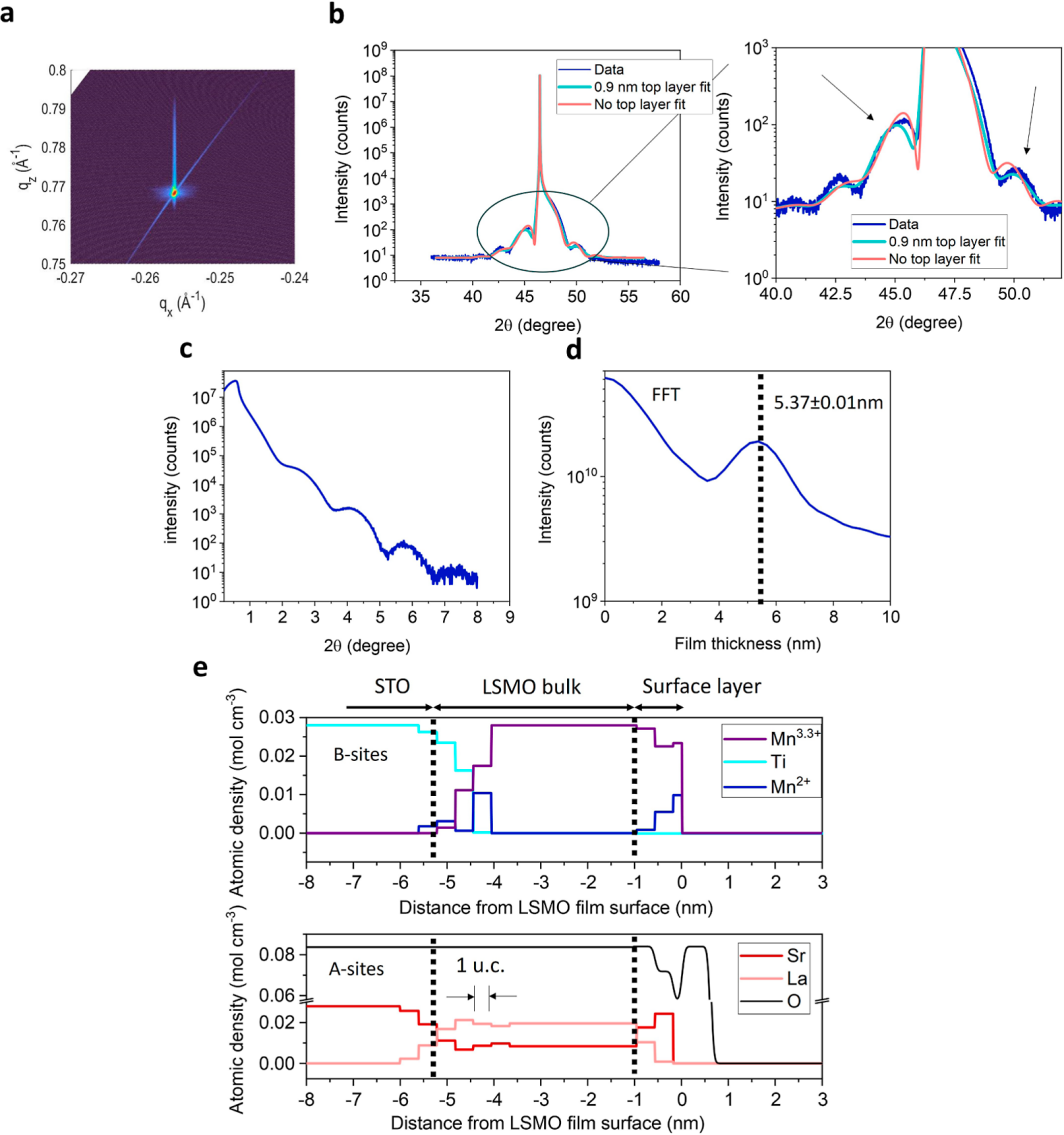
XRD and RXR analysis of the 13 u.c. LSMO film. (a) Reciprocal space map around the STO 103 peak. (b) XRD 2Θ–ω scans around the STO 002 peak along with dynamical simulated diffactograms. The simulations were performed without or with a lower crystalline layer of 0.9 nm. The large deviations of the fringe at 42° are probably induced by the broadening of the experimental substrate peak, which is not taken into account in the simulations. Simulations were done with the Stephanov X-ray server.^40^ The arrows indicate large deviations from the data for the simulation without a top layer. (c) XRR data for 13 u.c. LSMO film. (d) The Fourier transform of c, which indicates a LSMO film thickness of 5.37 ± 0.01 nm. (e) Atomic depth profiles of La, Ti, Sr, Mn^2+^, Mn^3.3+^, and O obtained using RXR at 300 K. (a,e) adapted from ref (9).

Further XRD analysis on the LSMO 002 peaks was performed to investigate the crystallinity and strain state of the thin film. The reciprocal space map around the STO 103 peak in Figure 2a confirms that the bulk of the film exhibits coherent tensile strain. The clear Laue fringes in Figure 2b indicate a good crystallinity in the bulk of the film. However, comparing these measured fringes with an expected diffractogram of a 13 u.c. thick film, see Supporting Information S1, indicates a measured film thickness below 13 u.c. Furthermore, the measured crystalline layer is thinner than the thickness obtained from the Fourier transform of the XRR data in Figure 2c,d of 5.37 ± 0.01 nm, which is close to the expected value for a 13 u.c. thin film. This indicates, as discussed in more detail below, that a layer of low crystallinity but with an electronic density similar to that of the LSMO bulk is present. See Methods for the modeling details of the crystallinity variability in the film. Correlating this with the resonant X-ray reflectivity (RXR) results, see below, indicates that this layer of lower crystallinity is positioned at or near the surface. The existence of such a layer is further confirmed by the asymmetric period around the STO 002 peak, which indicates asymmetry in the film. The data can be better fitted when a layer of approximately 0.9 nm with low crystallinity is considered on the film surface, as shown in Figure 2b.

Due to the crystalline dissimilarity between the bulk and the surface, we turned to RXR to study the chemical disorder.^41^ With RXR, subnanometer depth-resolution of the elemental composition can be obtained.^42^ The depth profile of the LSMO film shown in Figure 2e indicates nonstoichiometric regions near the substrate/film interface and the film surface. At the buried interface, the intermixing of the STO substrate with the LSMO film persists for more than 1 nm, inducing a La/Sr ratio that deviates from the stoichiometric value of 2:1. The nonstoichiometry at the buried interface is a likely cause of the reported magnetic dead layers of LSMO.^43^ Furthermore, we observe Mn^2+^ at the buried interface, which could be an effect of the intermixing of Ti^4+^ atoms. Near the surface, we observed Sr segregation and La deficiency and the formation of Mn^2+^ species. The latter could be induced by the presence of oxygen vacancies in the surface layer.^32,44^ Surface nonstoichiometry is unlikely to be introduced by beam damage because with repeating scans no changes in stoichiometry were noted. On the basis of these results, the nonstoichiometry in the surface of the film could be a reason for the lower crystallinity in the surface compared to that of the bulk.

### Surface Imaging

**Figure 3:**
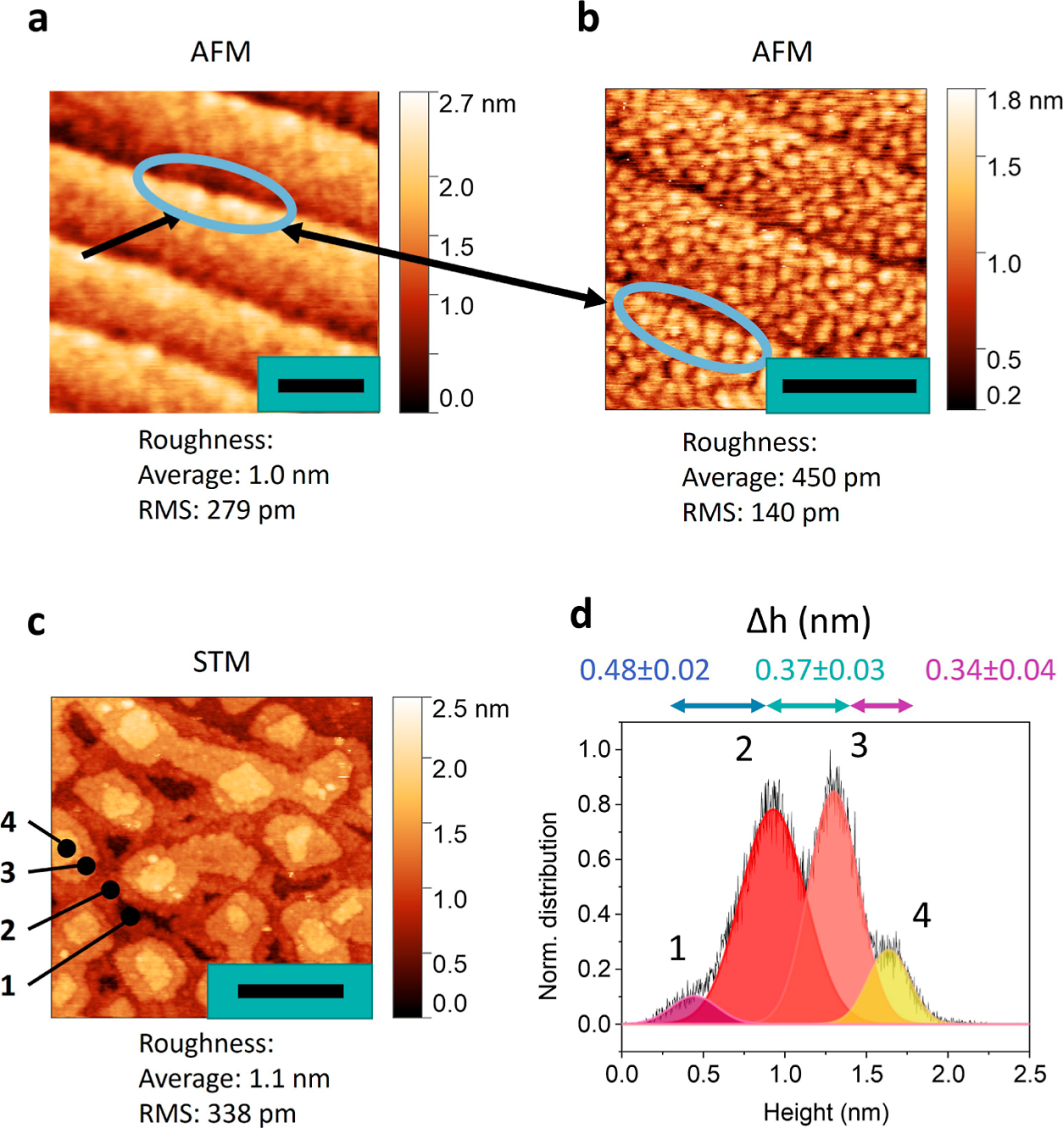
Surface structure of 13 u.c. LSMO thin films. (a) AFM imaging with a 30 nm radius tip showing mostly contrast from the step edges. The scale bar is equal to 100 nm. (b) AFM image with a tip radius of 5 nm indicating a granular surface morphology. Scale bar equals 40 nm. (c) High-resolution scanning tunneling microscopy (STM) image (800 mV, 100 pA) revealing a staggered growth. Numbers indicate consecutive layers. The scale bar is equal to 10 nm. (d) Height distribution of c with the peak spacing indicating the stacking of layers.

From the X-ray analysis, we identified that the surface of our LSMO films deviates from the high-crystalline bulk. We turned to atomic force microscopy (AFM) to study the surface structure of 13 u.c. LSMO films on STO in further detail. First, we imaged the surface with a rather large diameter tip. Our custom AFM probe has a pyramidal tip^45^ with a radius of around 30 nm, similar to commercial Si cantilevers. We used frequency-modulation (FM-AFM) feedback in repulsive mode. In this mode, the tip gently touches the sample and is lifted again during each oscillation cycle, similar to tapping-mode AFM. The benefit of this approach is that the AFM is most sensitive to short-range forces, increasing the resolution.^46^ However, the obtainable resolution is mainly limited by the radius of the tip.^47^ See Methods for further experimental details. As expected from the vicinal cut STO, the epitaxial thin film LSMO demonstrated flat plateaus and step edges, as shown in Figure 3a. These stepped film surfaces have often been reported in the literature for a wide range of transition metal oxide perovskites and layer thicknesses.^48,49^ The steps give rise to contrast in the AFM due to the convolution of the tip and the lateral friction when the side of the tip hits the atomic edge.^50^ To show that this contrast emerges from repulsive force or friction, even in FM-AFM when the side of the tip hits the step edge, we performed contact AFM. The topographic results are given in Supporting Information S2, where a clear contrast near the edge of the step is observed. Hence, step edges can dominate the AFM signal due to tip–sample friction during contact, and morphological details of the plateau surfaces can be overlooked. The conformation of the surface is slightly bulged upward near the step edge in Supporting Information S3. However, with the resolution obtained here, only a faint hint at nanometer-scale features of the scale of less than 1 u.c. height variation could be identified, as highlighted with the black arrows.

In order to improve lateral imaging resolution, we switched to a diamond tip with a radius of 5 nm; see Methods. Figure 3b shows a completely different surface morphology despite it being the same sample as that in Figure 3a. The surface is densely populated with semispherical objects with an average roughness of 450 pm and a mean roughness (*S*_a_) of 140 pm. These features are more closely packed and slightly taller near the step edge, as highlighted in blue, which explains why AFM with a lower imaging resolution of Figure 3a showed bulging toward the step edge.

To further explore the nanoscale surface structures of the LSMO films, we used STM in a UHV-SPM setup. The tip had a radius of less than 1 nm and the STM was mainly sensitive to electronic or height variations on the atomic scale. The STM image in Figure 3c showed that the semispherical structures are actually small stacked islands. These morphologies are known as staggered growth, or “wedding cake” islands.^30^ Similar features were observed for Ca-doped manganite^34^ and are termed mounds.^29^ Our tip-radius-dependent imaging highlights that, without using ultrasharp SPM tips, nanoscale features may be hidden and the surface morphology inaccurately is interpreted as atomically smooth on a scale of the width of the STO plateaus.

In Figure 3c, the most deeply buried layer, as indicated by number 1, is coalesced with only a few voids remaining in the area. We observe up to three layers stacked on top of this coalesced layer in the form of “wedding cake” islands or staggered growth, as shown numerically in the figure. By analyzing the statistical distribution of the height data, Figure 3d, we observe a combination of four height distributions. Each peak corresponds to a higher positioned layer in Figure 3c. We conducted a Gaussian fit to identify the peaks, as indicated by the colored curves. These peaks are not evenly spaced, as indicated by the numbers corresponding to the increase in the height (Δ*h*) of each stacking layer. However, they are close to the values for the thickness of 1 u.c. (0.385 nm) except for the layer numbered 1 to 2. This could be due to the fact that the area of the layer 1 is almost completely coalesced, so the convolution of the tip in the few small gaps has a negative effect on the accurate measurement of the thickness of the film. In this work, we observe clear layer-by-layer growth but no half-integer heights, which is different from the work of ref (29), where a peak spacing around 200 pm was observed indicating a mix of surface termination growth.

The variation in the peak-to-peak height of staggered growth can be as large as 2.5 nm, as can be seen in the maximum height value in Figure 3c. These large heights originate from individual dispersed spherical-like features across the surface. The film has an average roughness of 1.1 nm and an RMS roughness of 338 pm. With a film thickness of 13 u.c., this average roughness accounts for approximately 20% of the total film thickness. Based on the XRD results of Figure 2, we observed the thickness of the surface layer with low crystallinity in the 13 u.c. LSMO film was approximately 0.9 nm. This corresponds rather well with the observed staggered growth average film roughness by the STM and the off-stoichiometry of 2.5 u.c. (∼1 nm) from RXR.

The presence of staggered growth on the surface of the LSMO film was also observed for thicker layers (50 u.c.) grown on LaAlO_3_ (LAO) substrates, as shown in Supporting Information S4. LAO substrates introduce compressive strain in LSMO films, and thus the strain itself likely does not influence the occurrence of staggered growth to a large extent. Furthermore, a thicker LSMO film (50 u.c.) grown on STO shows similar corrugated surface features, as observed with AFM, in Supporting Information S4. Therefore, we conclude that the staggered growth of our films is an intrinsic property and important to consider.

To relate the observed surface structures to bulk crystallinity, we propose a two-step growth process. First, the deposited species during PLD nucleate and grow, manifesting themselves as spherical features, as observed in the SPM images. These spherical features move across the surface by diffusion and lateral aggregation but are impeded near the edges of the vicinal step due to the Erlich–Swoebel (ES) barrier.^30^ This barrier near the step edge impedes downward diffusion of surface features, leading to a densely packed morphology of islands toward the step edge region. Consequently, just below the step edge, there is a material deficiency, and few staggered structures can form there. We argue that the spherical features themselves are not necessarily an integer number of atomic u.c. stackings of the ABO_3_ perovskite. However, after embedding the features into a staggered growth layer, the thickness converges to integer u.c. heights, Figure 3d, likely driven by crystal restructuring. The disorder in the morphology of the staggered growth breaks long-range crystallinity and explains why a thinner fully crystalline bulk thickness is obtained, in comparison to the total film thickness by XRD fitting. Possibly, Sr segregation or oxygen vacancies^44^ break the stoichiometry of the islands, as observed with RXR in Figure 2e. The spherical nature of the islands is further identified by FFT filtering of the STM data in Supporting Information S5. When features associated with the staggered growth are removed, the spherical features remain and can be clearly observed. During PLD, these spherical features are formed on top of the islands and experience another ES barrier at the periphery of the island below. The coalescence and growth of approximately circular islands are naturally accompanied by gaps across the layers. As the islands expand in width, the gaps gradually coalesce. Only when these islands have reached a sufficient size can second layer nucleation occur.^51^ Consequently, we did not observe the nucleation of smaller islands or spherical features on nearly all of the highest islands. Rather rare in presence, we sometimes observed small individual spherical features scattered throughout the surface, as seen in Supporting Information S6. This observation is consistent with the work of Tershoff et al.^52^ In their work, it was shown that nucleation of a second layer occurs only when an island has reached a critical diameter in combination with a sufficiently strong ES barrier of the island boundaries. This seems to imply that before a critical size is reached, surface species can diffuse through the ES barrier and contribute to an increase in the lateral dimension of the islands. Only when a critical size has been reached is the ES barrier significant in magnitude to block species that diffuse downward and form a new layer of staggered growth. The second step of the film growth process occurs after coalescing of a fully closed layer. We argue that reconstruction can occur in these closed layers, forming a crystalline bulk, as seen in the XRD data. However, local defects could still persist,^29^ leading to a chemical dopant induced disorder.^20^

### Variation in Surface Electronic Structure

**Figure 4:**
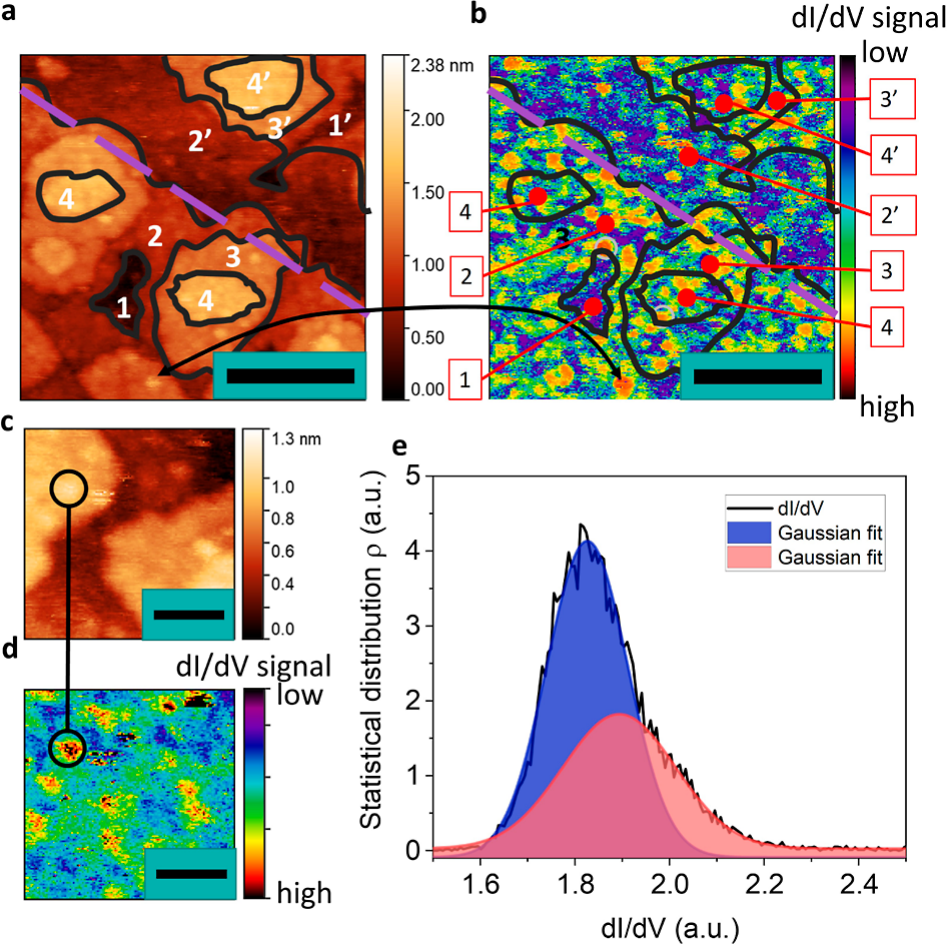
STS on 13 u.c. LSMO thin film demonstrating EPS. (a) Topography STM image (800 mV, 100 pA) with numbers indicating staggered growth islands. The purple dashed line indicates the step edge. The numbers 1′–4′ are layers located on an STO plateau below the step edge. The scale bar is equal to 10 nm. (b) Corresponding d*I*/d*V* map at 800 mV. The scale bar is equal to 10 nm. (c) Small-scale topography (800 mV, 100 pA) and (d) corresponding d*I*/d*V* map. The scale bar is equal to 2 nm. The black circle highlights the increased LDOS of a circular feature observed in the topography. (e) Statistical d*I*/d*V* distribution of d fitted with a Gaussian and showing two distributions of LDOS.

The local electronic structure of the complex morphology of the LSMO surface was studied in more depth. The presence of electronic disorder or EPS was investigated by STM and STS, respectively. Figure 4a,b shows the topography and simultaneously mapped d*I*/d*V* or local density of states (LDOS) measured at 800 mV at 300 K, respectively. For the d*I*/d*V* map, the areas in red have a larger LDOS than those in blue. Staggered growth island contours are highlighted with black lines and numbered at each consecutive stacked layer. From the d*I*/d*V* map, we observe that the layer, indicated with **1** in Figure 4b is rather homogeneous in LDOS with only some smaller local LDOS spots. This layer is almost structurally coalesced with a few remaining voids. However, the staggered growth layers 2–4 show a greater variation in the intensity of LDOS. The d*I*/d*V* signal can be correlated with the presence of surface spherical features, as highlighted by the black circles in Figure 4c,d. These spherical features possess a larger LDOS, and hence it is likely that they possess a nonstoichiometric chemical composition, with an excess of Sr making the spherical features more conductive. These spherical features are rather dispersed throughout the staggered growth.

The EPS persists down to the nanometer scale. In Figure 4c,d, a 10 × 10 nm image reveals a clear inhomogeneity of d*I*/d*V* with some electronic domains less than 1 nm in diameter. A d*I*/d*V* spectrum of a 50 u.c. LSMO grown on LAO is shown in Supporting Information S7, which does not show an indication of a large insulating gap in de LDOS. During growth, Sr adatoms may diffuse through the staggered growth, giving rise to a local variation in LDOS. Such surface roughness that can be accompanied by EPS has previously been reported for Ca-doped LMO.^34^ Below the step edge, as indicated by the purple dotted line, Figure 4a,b, a small region numbered 2′, of less than 10 nm in width, is observed. It has little staggered growth and rather uniform LDOS, showing that the surface morphology and the EPS are correlated for the LSMO thin films.

The distribution of the d*I*/d*V* map of Figure 4d indicates a bimodal distribution, as shown in Figure 4e. This distribution is likely an indication of the chemical disorder caused by the local excess Sr and oxygen vacancies discussed previously. Similar electronic inhomogeneities were observed for thicker LSMO films (50 u.c.) grown on LAO substrates (compressive strain in LSMO), given in Supporting Information S4, confirming the relation with staggered growth on the surface. A possible effect of this disorder is magnetic inhomogeneity due to the local variation in chemical composition. This magnetic inhomogeneity is important to consider because of the spin-ordering effect in the OER.^9^

**Figure 5:**
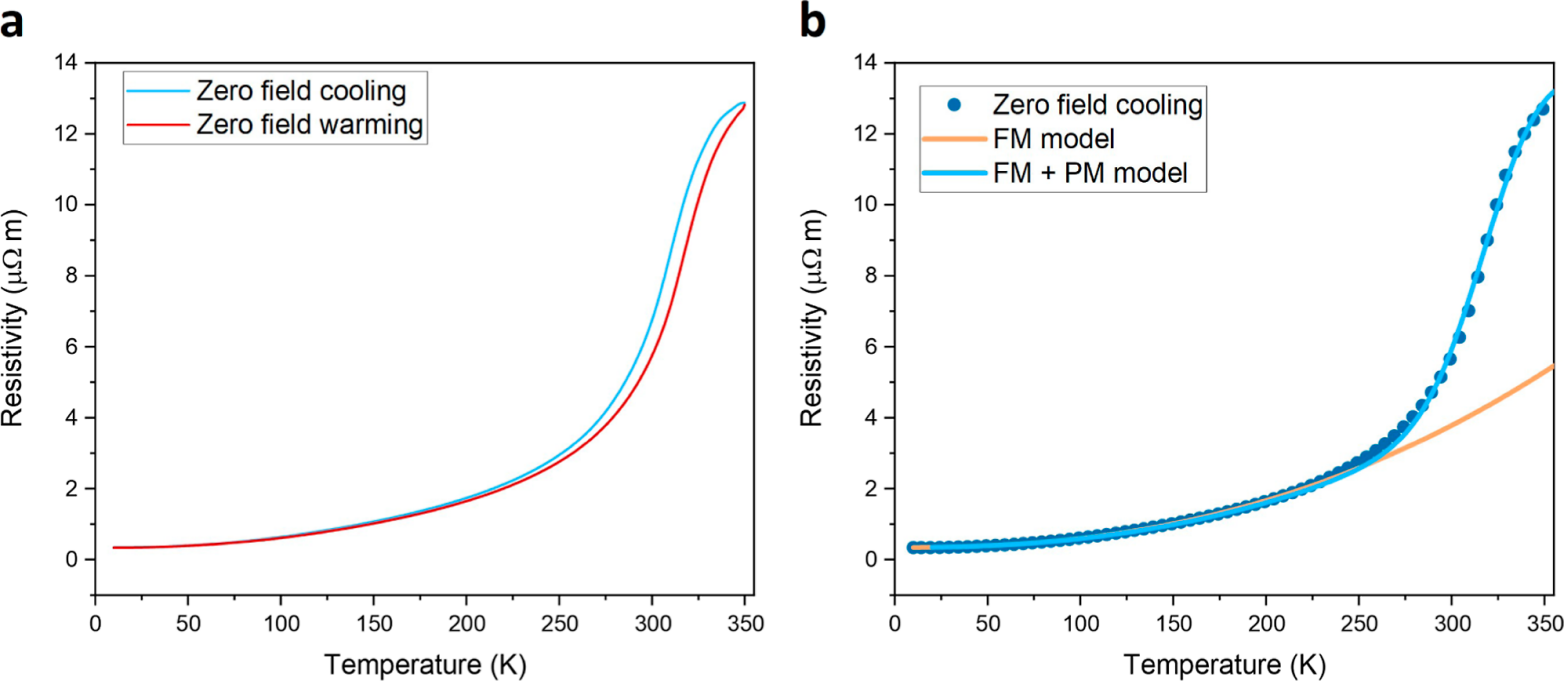
Zero field cooling and warming of the resistivity behaviour. (a) Zero field cooling and warming of a 13 u.c. LSMO thin film with hysteresis observed near and above room temperature. (b) Fitting a resistivity percolation model of phase-separated FM and PM clusters (blue curve)^35−37^ and a FM model (orange).^35−37^

To further verify this, we attempted to correlate a possible microscale magnetic inhomogeneity with bulk transport measurements, which has been done before for manganites^20,22^ and relates percolation theory^53^ to the emergence of a nonlinear response such as magnetoresistance. In our work, we observed two nanoscale LDOS phases. In the percolation theory of phase separation, this electronic disorder introduces hysteresis or memory effects in zero field cooling and warming of the resistivity *R*(*T*). The results in Figure 5a indicate such a hysteresis between zero field cooling and zero field warming cycle. The temperature dependence of the resistance can be regarded as a system in competition between FM metallic areas, with a long spin correlation length, and PM areas with a vanishing or very small spin correlation length. For Sr- and Ca-doped manganites, a phenomenological model was developed in refs (35–37) that describes the temperature dependence of the resistance as a percolation between a volume fraction of the PM and FM areas. Here, we fit the same model for LSMO. In this model, the metallic conductivity is described with an electron–electron interaction by an *AT*^*N*^ term. Therefore, the FM resistance can be given as ρ_FM_(*T*) = ρ_0_ + *AT*^2.35^ with *N* obtained from fitting. For high temperatures, above *T*_c_, the resistance can be described using a magnetic polaron picture^35−37^ represented as , with *E*_g_ the activation energy and *k*_b_ the Boltzmann constant. We fit this phenomenological model to the *R*(*T*) data, and a reasonable agreement is shown in Figure 5b. For intermediate- and high-temperature regions, the FM + PM model agrees well with the data. We fitted the low-temperature regime with only the FM model, and good agreement was reached, as indicated by the orange line in Figure 5b. On the basis of these results, we conjecture that the observed structural and EPS/disorder of LSMO epitaxial thin films is likely correlated with the macroscopic film resistive behavior and complex FM and PM phase separation occurs, especially at elevated temperatures.

## Conclusions

The combination of XRD and RXR characterization and SPM imaging revealed a complex surface and electronic morphology of LSMO thin films grown on STO by PLD. Using element-specific RXR, we revealed a gradient in the chemical composition of the film that showed a nonstoichiometric top layer of around 1 nm with an excess of Sr, oxygen vacancies, formation of Mn^2+^ valence states, and a La deficiency. This chemical disorder was further studied on the nanoscale with SPM where we revealed a staggered growth on the surface of the films consisting of nanoscale-stacked islands and composed of spherical features. The formation of staggered growth was described by a two-step growth model driven by an Ehrlich-Schwoebel barrier on the surface and a reconstruction of the bulk, yielding high crystallinity. We correlated the staggered growth with the formation of surface EPS/electronic disorder driven by nonstoichiometry and resulting magnetic inhomogeneities. For applications such as electrochemistry correlated to the fundamental theoretical understanding of the complex oxide manganites, careful consideration of the surface morphology and electronic structure is important. Therefore, the results presented in this work of the disentanglement between the bulk and surface properties of LSMO make it a tantalizing model system to evaluate the relationship between the surface and bulk spin ordering in electrocatalysis.^9,10,54,55^

## Methods

### PLD and RHEED

Thin films of La_0.67_Sr_0.33_MnO_3_ were fabricated by using PLD and a stoichiometric target from SurfaceNet. The vacuum system had a base pressure of 5 × 10^–8^ mbar and was equipped with an in situ RHEED and a KrF excimer laser of 248 nm. B-site terminated and step-terraced STO (001) substrates were purchased from CrysTec GmbH or Shinkosha Co. Ltd. To ensure close to ideal TiO_2_ termination of our STO substrates, we employ the substrate treatment as suggested by Koster et al. in ref (56) Due to the nature of this treatment, we assume that the substrates will be predominantly terminated with TiO_2_. The laser fluence was set to 2.0 J cm^–2^ and the frequency used was 1 Hz. The oxygen pressure was 0.266 mbar and the substrate temperature was 750 °C. The distance between the sample and the target was 5 cm and a rectangular mask was used to obtain a laser spot size of 2.24 mm^2^. The targets were preablated at 10 Hz to remove any possible surface contamination. After deposition, the samples were slowly cooled with a rate of 25 °C/min inside the PLD at 100 mbar oxygen pressure.

### X-ray Diffraction

XRD and reflectivity measurements were performed by using a Bruker D8 Discover diffractometer with Cu–Kα radiation and an Eiger2 R 500 K area detector. A Ge(022) monochromator was used for diffractograms, and a collimator of 1 mm diameter was used to obtain Cu Kα1 radiation and shape the incident beam. The detector was operated in 0D mode with a small region of interest (975 μm × 4575 μm) during the 2Θ–ω scans. To obtain the RSM, the detector was kept stationary while operating in 1D mode while an omega rocking curve was performed. Reflectivity measurements were made using a 0.1 mm slit and a 1 mm collimator to shape the incident beam. The detector was operated in 0D mode with a small region of interest (975 μm × 4575 μm). Comparison of the measured and expected diffractograms of the LSMO film peaks around the STO 002 peak in a 2Θ–ω scan of the XRD spectra was performed using InteractiveXRDFit software.^57^ More in-depth simulations of the XRD diffractogram in this study were carried out using the GID SL program at Sergei Stepanov’s X-ray Server.^40^ The parameters used to vary the crystallinity of the layer are the Debye–Waller like factors (*w*_0_ and *w*_h_), which form a correction to scattering and absorption factors based on the crystallinity of the material.^40^ Its value ranges from 0 to 1, where 0 is an amorphous layer, and 1 corresponds to a perfect crystal without defects. For the more crystalline layer, the values of *w*_0_ and *w*_h_ were taken as 0.85 to include a small number of defects and amount of disorder, typically present in PLD grown films. For the lower crystalline layer, a value of 0.75 generated the best fit. A value of 1 was used for *w*_0_ and *w*_h_ for the crystalline substrate. The roughness values used were equal to 2 Å for the substrate and 4.5 Å for the film, in accordance with the roughness measured by using AFM.

### Resonant X-ray Reflectometry

#### Data Acquisition

Data acquisition of the RXR was done at the resonant elastic and inelastic X-ray scattering beamline of the Canadian Light Source in Saskatoon, Canada.^41^ A flux of 5 × 10^12^ photons per second with a photon energy resolution Δ*E*/E of ∼10^–4^ was used for the measurements. Linear photon polarization was used with the electric field vector normal to the reflection plane and within the surface plane of the sample (sigma polarization). Measurements were done at a temperature of 300 K under a base pressure of 1 × 10^–9^ mbar. The reflection geometry scans were made possible by an in-vacuum four-circle diffractometer after the samples were aligned with their surface normal in the scattering plane. Measurements were taken at several resonant photon energies at different resonances: Ti L_2,3_ (∼450–470 eV), Mn L_2,3_ (∼635–660 eV), and La *M*_4,5_ (∼830–860 eV), along with multiple nonresonant photon energies. The measurements were done in a specular reflection geometry. A photodiode was used to detect the reflected beam intensity with the response function of the photodiode determined by directly measuring the synchrotron beam. All measured data were normalized by the incident beam flux and the response function to obtain the quantitative reflectivity spectra. The full RXR data set is presented in the Supporting Information.

#### Modeling

Global optimization of resonant X-ray reflectometry, a software package recently developed by the QMaX group at the University of Saskatchewan, was used for modeling of the RXR data. We used tabulated atomic form factors for nonresonant energies^58^ and measured X-ray absorption for the construction of resonant scattering tensors for elements Ti, Mn, and La. For Mn, we differentiated the resonant scattering tensors for Mn^2+^ and Mn in stoichiometric LSMO (implemented as a weighted linear combination of Mn^3+^ and Mn^4+^ scattering tensors corresponding to Mn^3.3+^). A slab model was used to construct the optical depth profile. Such a model is made up of parametrized layers with defined elements, oxidation states, roughnesses, thicknesses, and densities. By modeling the film at the u.c. level using the model parameters, we construct an element-specific, discrete, depth-dependent density profile. This so-called u.c. model fixed the thickness of each layer with a value that corresponds to the lattice constant of a theoretical LSMO/STO heterostructure and models the roughness as a step function at the edge of each u.c. A density profile of the 13 u.c. LSMO samples is determined by optimizing the density of the elements and oxidation states present in each u.c. layer. First, an approximation of the density profile, along with the form factors, is used to determine the expected energy- and depth-dependent optical profile. The optical profile is then used to simulate the reflectivity for a given energy, reflection angle, and polarization. Finally, we optimized the density by fitting the simulations to the experimental data. The concentration of Sr and La is fixed to their stoichiometric ratio throughout the bulk of the film to reduce the parameter set; however, it is allowed to vary at the interface and surface of the film. The overall elemental density profile was first determined by optimizing the parameters against the extended sigma-polarized experimental data set.

The RXR data set and associated fits of the Mn-resonant energy scans are shown in Supporting Information, Figure S8. The resonant and nonresonant theta/two-theta reflectivity scans at different energies and their fits are displayed in Supporting Information, Figure S9. Further details about the modeling are given in ref (9).

### Scanning Probe Microscopy

#### UHV Atomic Force Microscopy

AFM was performed with a Scienta Omicron GmBH UHV VT-SPM operating in ultrahigh vacuum with a base pressure of 10^–10^ mbar. A self-sensing quartz tuning fork (AB38T) was customized as a two-prong force oscillating sensor^45^ with a *Q*-factor of 15 000. Using a cleaved silicon wafer, a pyramidal-shaped tip is formed with a radius of about 30 nm. Frequency-Modulation AFM using constant frequency shift feedback was used with a phase-lock loop. The tip oscillated at an amplitude of 1 nm. The ultrasharp-tip AFM sensor was fabricated by using a diamond tip (AdamaProbe). This tip was glued to one prong of the self-sensing tuning fork using EPOTEK E2101 UHV compatible two-component epoxy. AFM was operated in the FM-AFM mode. The *Q*-factor of the sensor was around 40 000. The tip oscillated with an amplitude of 1.5 nm. Postprocessing of the AFM data was performed with Gwyddion software.^59^ Images were line aligned using the median of differences and plane-leveled using mean subtraction.

#### Ambient Atomic Force Microscopy

Contact-AFM was performed using a Nanosensor NCH-PPP Si cantilever on a Veeco Dimension III microscope in ambient. Imaging was carried out with a normal force of 5 nN with a scan speed of 1 line per second (512 lines/512 pixels). The friction image was obtained by registering the lateral movement of the cantilever detected by the four-quadrant photodetector.

#### UHV Scanning Tunneling Microscopy and Scanning Tunneling Spectroscopy

STM was performed with a Scienta Omicron GmbH UHV VT-SPM operating in ultrahigh vacuum with a base pressure of 10^–10^ mbar. STM tips were mechanically cut from a PtIr wire. The bias was applied to the tip, and the sample was grounded. The imaging was performed in a constant current mode. For d*I*/d*V* mapping and spectroscopy, an Oxford Instruments lock-in amplifier with an alternating voltage of 100 mV applied at 4.1 kHz was used. Postprocessing of the STM data was performed with Gwyddion software.^59^ The images were line-aligned using the median of differences and plane-leveled using mean subtraction. Before STM, the sample was exposed to ambient conditions, and surface contamination was excluded to significantly contribute to the d*I*/d*V* contrast. The SPM results presented in this paper were performed at 300 K.

## Supporting Information Available

Comparison of the measured and expected diffractogram of the LSMO film peaks around the STO 002 peak in a 2Θ–ω scan, topographic map with contact-mode AFM showing contrast predominantly from the step edges, line cross-section of topographic AFM map, STM and AFM on 50 u.c. LSMO grown on LAO and STO, FFT filtering of the LSMO surface, STM observation of individual spherical features, STS spectra of the surface growth, and RXR resonant theta/two-theta reflectivity scans and Mn resonant energy scans (PDF)
