# Supplementary material for: Electronic and Structural Disorder of the Epitaxial La0.67Sr0.33MnO3 Surface
Source: ACS Appl Mater Interfaces. 2024 Apr 15;16(16):21273–82. doi: 10.1021/acsami.3c17639 (PMC11056928; doi:10.1021/acsami.3c17639)
Supplement: Supplementary file 1 — am3c17639_si_001.pdf [file am3c17639_si_001.pdf]

Supporting Information - Electronic and structural disorder of the epitaxial $La_{0.67}Sr_{0.33}MnO_3$ surface

Michael Verhage,[†,#] Emma van der Minne,[‡,#] Ellen M. Kiens,[‡] Lucas Korol,[¶] Raymond J. Spiteri,[§] Gertjan Koster,[‡] Robert J. Green,[‖,¶] Christoph Baeumer,[*,‡,⊥] and Cornelis F.J. Flipse[*,†]

†*Molecular Materials and Nanosystems (M2N) - Department of Applied Physics - Eindhoven University of Technology, Eindhoven, Netherlands*

‡*MESA+ Institute for Nanotechnology, Faculty of Science and Technology, University of Twente, Enschede, Netherlands*

¶*Department of Physics & Engineering Physics, University of Saskatchewan, Saskatoon, Canada*

§*Department of Computer Science, University of Saskatchewan, Saskatoon, Canada*

‖*Stewart Blusson Quantum Matter Institute, University of British Columbia, Vancouver, Canada*

⊥*Peter Gruenberg Institute and JARA-FIT, Forschungszentrum Juelich GmbH, Juelich, Germany*

#*Contributed equally*

E-mail: c.baeumer@utwente.nl; c.f.j.flipse@tue.nl

S1

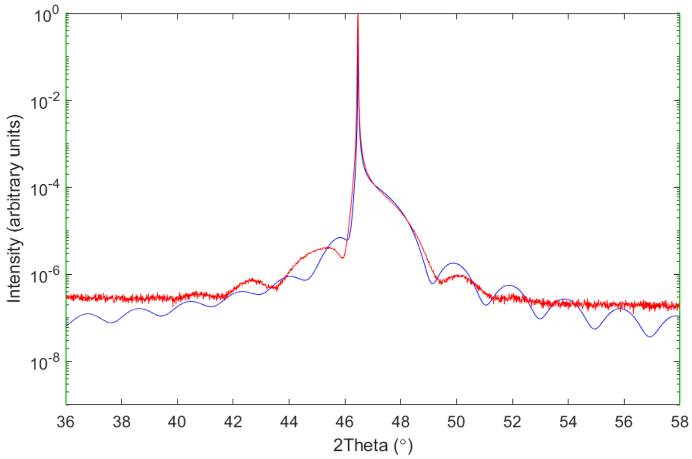

Figure S1: **Comparison of the measured (red curve) and expected diffractogram (blue curve) of the 13 u.c. LSMO film around the STO 002 peak in a 2Θ-ω scan**. The expected diffractrogram is obtained with the Interactive XRDFit software[1] using a film thickness of 13 u.c. and lattice parameter $c = 3.845$ Å.

S2

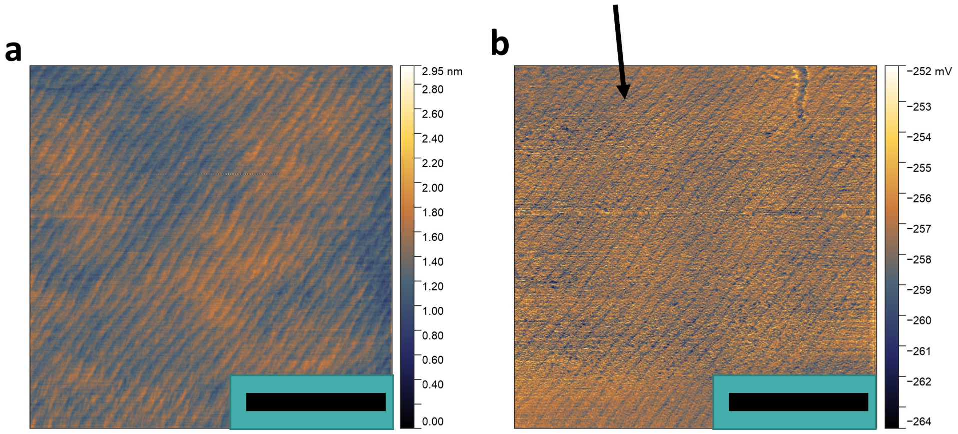

Figure S2: **Contact-mode AFM.** (**a**) Topographic map with contact-mode AFM showing contrast predominantly from the step edges. (**b**) Friction map shows stronger signal (blue) indicated along the step edges (indicated by the black arrow), while it shows little on the plateaus. The black scale bar is equal to 2 µm.

S3

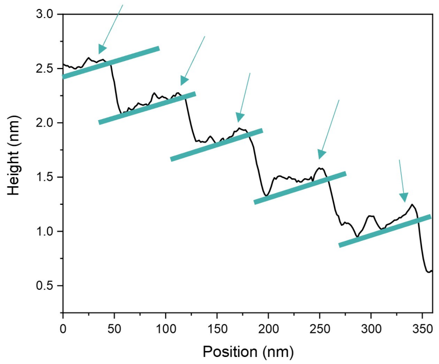

Figure S3: **Line cross-section of Figure 3a on the main text.** The green arrows indicate upward bulging of the topography near the step edge. The plateaus are indicated with the green lines.

S4

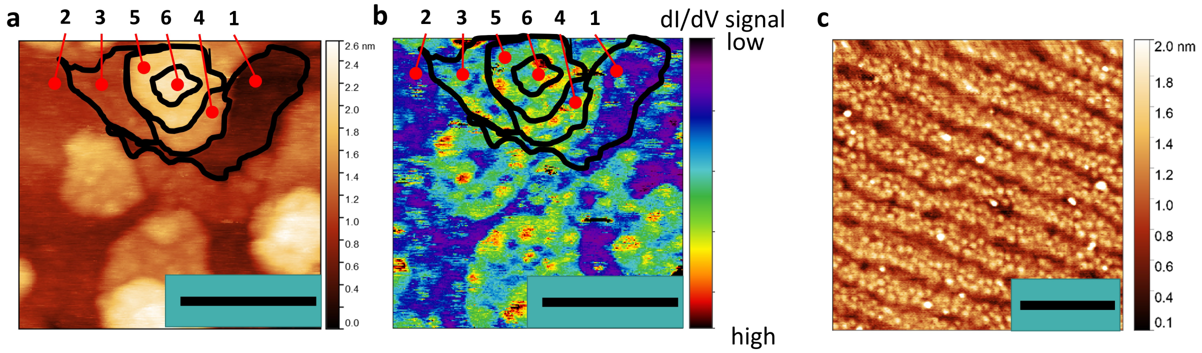

Figure S4: **Staggered growth of 50 u.c LSMO grown on $LaAlO_3$ (LAO) and $SrTiO_3$ (STO) single crystal substrates.** (**a**) STM image (1 V, 50 pA) of 50 u.c. LSMO on LAO indicating staggered growth islands highlighted with numerals. The black scale bar is equal to 10 nm. (**b**) Corresponding STS image indicating electronic inhomogeneity correlated to staggered growth. The black scale bar is equal to 10 nm. (**c**) Large scale AFM image showing stepped morphology of the STO substrate and circular features indicative of staggered growth of the LSMO film (50 u.c.). The black scale bar is equal to 500 nm.

S5

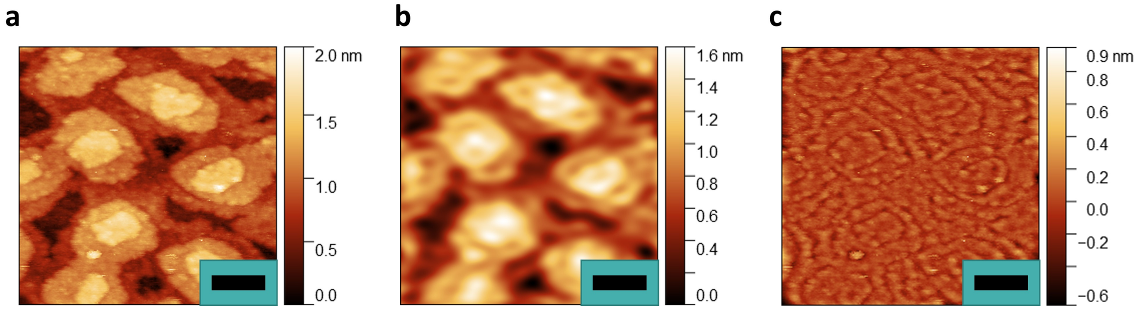

Figure S5: **FFT filtering showing spherical features across the staggered growth of the LSMO surface.** (**a**) STM image of 13 u.c. LSMO thin film. (**b**) Fast Fourier (FFT) filtering of the staggered growth. (**c**) After the removal of the staggered growth by FFT filtering, leaves only the spherical features of the film surface highlighted. The black scale bar is equal to 5 nm.

S6

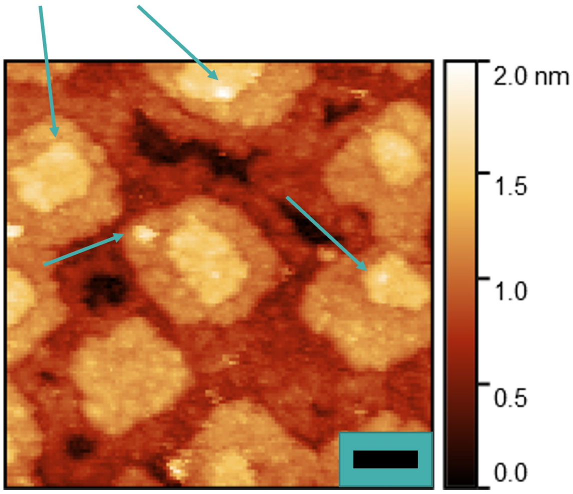

Figure S6: **STM observation of individual spherical features.** STM image of 13 u.c. LSMO/STO film with staggered growth showing individual spherical features distributed ontop of the upper layers, as indicated with the arrows. The scale bar is equal to 5 nm.

S7

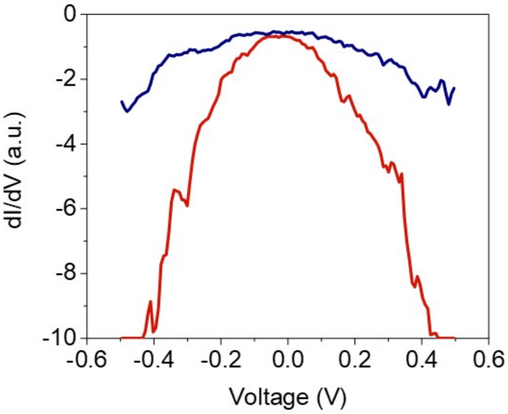

Figure S7: **STS spectra showing local LDOS variation of a 50 u.c. LSMO film on LAO substrate.** The blue and red STS curve correspond to the flatter and staggered growth regions of the LSMO surface, respectively.

S8

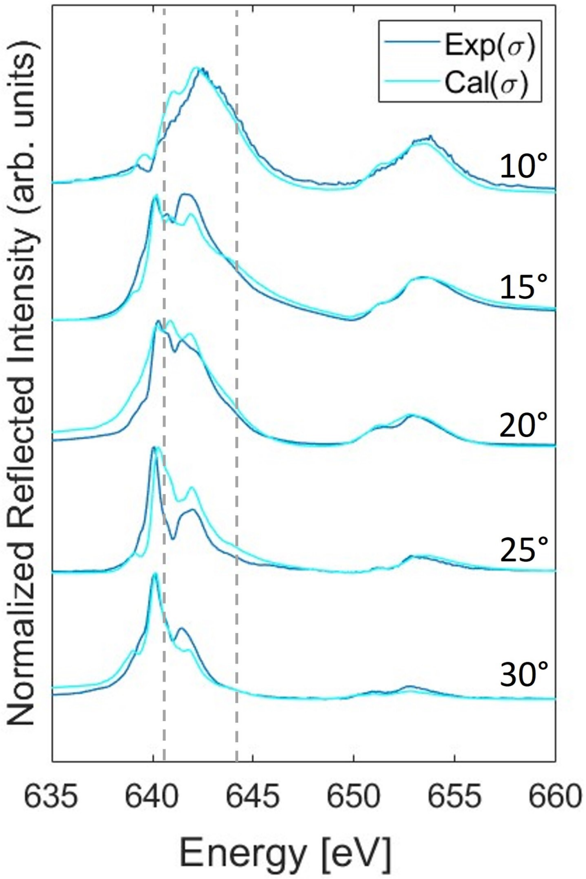

Figure S8: **RXR additional data**. Mn-resonant energy scans of a 13 u.c. $La_{0.67}Sr_{0.33}MnO_3$ film on $SrTiO_3$ measured at a temperature of 300 K. Vertical dashed lines indicate the energy for the non-resonance scans in S9, Figure S9. The experimental (exp) and simulated data (calc) pairs are included in each plot providing a label for each pair's corresponding grazing angle. The results were normalized and each set of curves is offset vertically for clarity.

S9

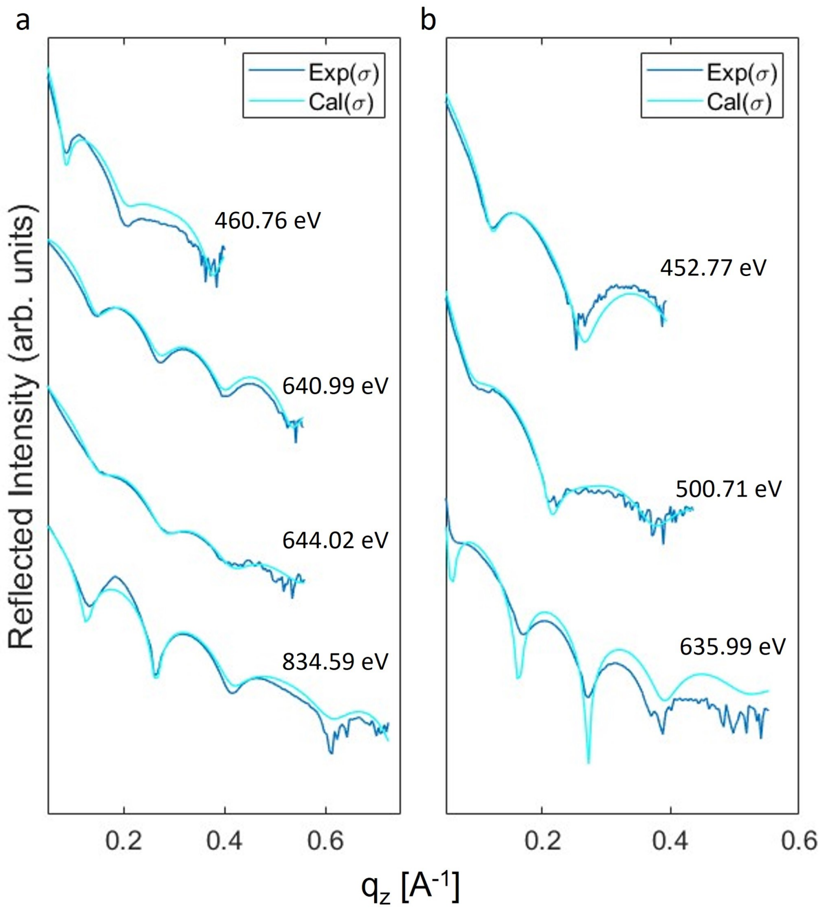

Figure S9: **RXR additional data (a)** Resonant theta/two-theta reflectivity scans of a 13 uc $La_{0.67}Sr_{0.33}MnO_3$ film on $SrTiO_3$ measured at a temperature of 300 K. **(b)** Non-resonant theta/two-theta reflectivity scans of the same 13 u.c. film measured at a temperature of 300 K. The experimental (exp) and simulated data (calc) are included in each plot, where each pair is labeled with their corresponding photon energy. Some of the chosen photon energies are indicated. Each set of curves is offset vertically for clarity.

References

(1) Lichtensteiger, C. *InteractiveXRDFit* : a new tool to simulate and fit X-ray diffractograms of oxide thin films and heterostructures. *Journal of Applied Crystallography* **2018**, *51*, 1745–1751.
